# Diffuse histology-proven mucinous cystic neoplasm of the pancreas: A case report and review of literature

**DOI:** 10.1016/j.ijscr.2019.10.015

**Published:** 2019-10-15

**Authors:** Clara T. Nicolas, Sameer Al Diffalha, Sushanth Reddy

**Affiliations:** a Department of Surgery, University of Alabama at Birmingham, Birmingham, AL, United States; b Department of Pathology, University of Alabama at Birmingham, Birmingham, AL, United States

**Keywords:** Mucinous cystic neoplasm, Pancreas, Pseudocyst, Case report

## Abstract

**Introduction:**

Mucinous cystic neoplasms (MCN) are rare premalignant neoplasms of the pancreas that are typically found as single lesions in the pancreatic body and tail of women in the fifth and sixth decade of life, do not communicate with the pancreatic ductal system and are characterized by mucin-producing epithelium supported by ovarian-type stroma.

**Presentation of case:**

We present here a case of diffuse pancreatic involvement by MCN in a 64-year-old woman with chronic pancreatitis. Pre-operative suspicion for MCN was low due to the multi-centric nature of the lesions and imaging/biochemical fluid analysis demonstrating connection with the pancreatic ductal architecture. The patient underwent total pancreatectomy with pathology showing multiple cysts lined by flat epithelium with focal ovarian-type stroma, consistent with low-grade MCN.

**Discussion:**

The presence of ovarian stroma on histological analysis is one of the defining characteristics of MCNs per WHO guidelines, and is mandatory for its diagnosis. Only one case of diffuse MCN has been previously described in the literature; however, in this case the authors were not able to reach a definitive histological diagnosis based on WHO criteria.

**Conclusion:**

To our knowledge, this is the first report of diffuse histology-proven MCN of the pancreas.

## Highlights

• MCN is very rarely multicentric.
• Presence of ovarian-type stroma is required for the diagnosis of MCN.
• This is a case of diffuse MCN in the setting of chronic pancreatitis.
• It highlights the importance of multidisciplinary care for pancreatic cystic disease.

## 1 Introduction

Mucinous cystic neoplasms (MCN) of the pancreas are rare tumors, constituting 2–5% of pancreatic neoplasms, that are found almost exclusively in perimenopausal women as single lesions in the body and tail of the pancreas with no connection to the ductal system [1]. Due to their premalignant nature, the recommended treatment for MCNs is resection, usually by means of a distal pancreatectomy. They are very rarely multifocal, and it is unclear whether the risk of malignancy increases with more than one lesion.

Although multifocal disease is common in the setting of other pancreatic cystic lesions, such as intraductal papillary mucinous neoplasms (IPMNs), only a handful of cases involving more than one synchronous MCNs have been described. The two entities were often confused until the World Health Organization (WHO) proposed the presence of ovarian stroma on histological analysis as one of the defining characteristics of MCNs, making it mandatory for its diagnosis [2]. We present here the first case, to our knowledge, of diffuse histology-proven pancreatic MCN. The work reported is in line with SCARE guidelines [3].

## 2 Case report

A 64-year-old female was referred to our surgical oncology clinic for evaluation of cystic lesions of the pancreas found on CT scan at an outside hospital after she presented with a history of approximately nine months of vague epigastric pain, nausea and vomiting. She had a 50-year history of daily tobacco use (0.5 pack per day) and nightly alcohol use that had reportedly stopped around the same time as the onset of her symptoms, but she was otherwise healthy.

**Fig. 1:**
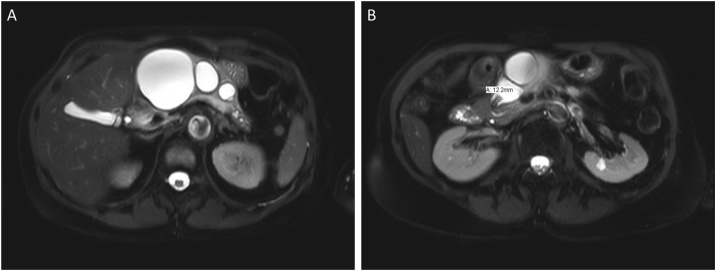
MRCP showing innumerable saccular dilations (A) associated with dilation of the main pancreatic duct and a 1.2 cm stone in the pancreatic head (B).

**Fig. 2:**
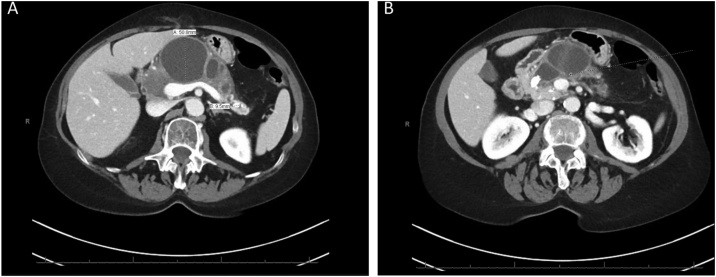
CT abdomen showing multiple cystic lesions within the pancreas. The largest cystic lesion in the mid-body pancreas measures 5.1 cm in maximal diameter and appears to communicate with the main pancreatic duct (A). Also seen is a cluster of smaller cystic lesions within the pancreas head and tail. Downstream, again seen is a large 1.7 cm pancreatic duct calculus (B). The head and uncinate process of pancreas demonstrate multiple scattered punctate calcifications.

She had also undergone an MRI/MRCP at the outside hospital that revealed multiple cystic loculations of the pancreas that appeared to be possibly in communication with the main pancreatic duct (Fig. 1). There were calcifications in the head of the pancreas consistent with chronic pancreatitis. Her Carbohydrate Antigen 19-9 (CA 19-9) was <1. A three-phase pancreas protocol CT scan was performed at our institution that showed dilation of the main pancreatic duct with innumerable saccular cystic dilations, thought to be likely dilated side branches arising from the pancreatic duct (Fig. 2). The working diagnosis was the presence of pancreatic pseudocysts in relation to chronic pancreatitis. Her local gastroenterologist proposed endoscopic transgastric cystic drainage of her dominant cyst. After evaluation at our institution, the differential diagnosis included pseudocysts secondary to chronic pancreatitis as well as diffuse, multiple intraductal papillary mucinous neoplasms (IPMN).

The patient underwent an endoscopic ultrasound with fine needle aspiration and cyst fluid analysis, demonstrating a Carcinoembryonic Antigen (CEA) level of over 600 ng/mL, an amylase level of over 75,000 U/L, and rare clusters of mucinous epithelium, consistent with a mucinous tumor communicating with the pancreatic ductal system. Her case was discussed at our institution’s Pancreaticobiliary Disease Center Tumor Board and total pancreatectomy was recommended given the extent of these mucinous tumors and high suspicion for multifocal branched duct IPMN.

The patient underwent a total pancreatectomy with duodenectomy, cholecystectomy, and splenectomy, and creation of a choledochojejunostomy and gastrojejunostomy. Intraoperatively, there was extensive scarring in the pancreatic head consistent with a history of pancreatitis, and the patient’s diffuse multicystic disease was immediately apparent upon visualizing the pancreas. The operation proceeded as planned and the patient was admitted to the surgical floor, where she demonstrated an uncomplicated postoperative course. She was discharged home on postoperative day seven with adequate glucose control.

**Fig. 3:**
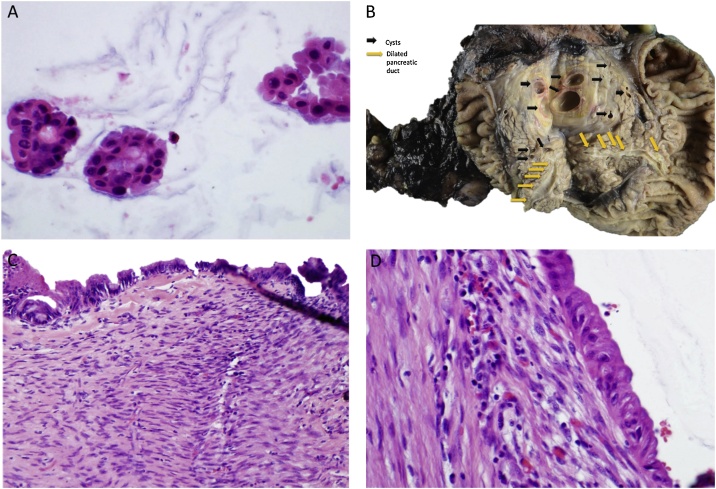
Cytologic, macroscopic, and histologic analysis. Fine needle biopsy shows rare clusters of mucinous epithelium (A). The pancreas is opened to reveal multiple cystic structures (ranging from 0.5 to 7.7 cm) containing a brown-tan, thin liquid located throughout the pancreatic head, neck, and tail. The cysts are surrounded by areas of fibrosis and contain multiple yellow-tan to white-tan stones. The cysts involve ∼80% of the pancreatic parenchyma and obstruct the pancreatic duct at the pancreatic neck (B). The entire cystic lining was submitted for histologic exam and reviewed. Sections show cysts lined by flat low-grade foveolar-type and focal pancreatico-biliary type epithelium with focal ovarian-type stroma (C & D), consistent with a low-grade mucinous cystic neoplasm. Many areas of the cyst are denuded and lined by granulation tissue with fibrinous exudate, cholesterol cleft and calcifications, with extensive surrounding fibrosis.

Macroscopically, the pancreatic specimen was opened to reveal multiple cystic structures located throughout the pancreatic head, neck, and tail, involving approximately 80% of the pancreatic parenchyma, and ranging from 0.5 to 7.7 cm in size. The cysts contained a brown-tan, thin liquid as well as multiple yellow-tan to white-tan stones, and were surrounded by areas of fibrosis. Microscopically, they were lined by flat low-grade foveolar-type and focal pancreaticobiliary-type epithelium with focal ovarian-type stroma, consistent with low-grade mucinous cystic neoplasm (Fig. 3). Many areas of the cysts were denuded and lined by granulation tissue with fibrinous exudate, cholesterol cleft, and calcifications with extensive surrounding fibrosis, consistent with chronic atrophic pancreatitis and pseudocyst formation. Several of the pseudocysts were in communication with a dilated main pancreatic duct. Ovarian stroma was present in the larger cysts. The duodenal, gastric, and common bile duct margins were negative. Twenty-eight lymph nodes were analyzed that were all negative for malignancy.

The patient is currently seven months out from her total pancreatectomy and is able to maintain her nutrition and hydration orally. She takes 48,000 units of pancrelipase with every meal and 24,000 with every snack. At her last clinic visit, she was on nine units of insulin glargine nightly and one to five units of sliding scale insulin lispro as needed, and she is being followed by an endocrinologist for glucose control optimization.

## 3 Discussion

Premalignant lesions of the pancreas include MCNs and IPMNs. Both are cystic, mucinous neoplasms that share many common features, and the distinction between the two was not made until the last two decades. According to WHO criteria [2], MCNs are composed of columnar, mucin-producing epithelium supported by ovarian-type stroma, ectopically incorporated into the pancreas during embryogenesis, and show no communication with the pancreatic ductal system. They occur almost exclusively in women, usually middle aged, are rarely seen in the pancreatic head and are even more rarely multicentric. IPMNs are composed of papillary, mucin-producing epithelium with no ovarian-type stroma, and arise in the main pancreatic duct or its major branches. They are more common in males than females and are found throughout a broad age range. Although IPMNs favor the pancreatic head, they can be seen throughout the pancreas, and diffuse involvement of the pancreas has been described. MCNs represent only 2–5% of all exocrine pancreatic tumors, while IPMNs represent 1–3%. Incidence of both of these tumors is growing with the advent of high-resolution abdominal imaging [4].

The 2004 International Association of Pancreatology (and later the WHO) mandated the presence of ovarian stroma in the diagnosis of MCN [5]. Some have suggested endodermal stimulation of immature stroma by estrogen and progesterone or primary yolk sac implantation in the pancreas as the etiology of MCN in the pancreas [6]. The buds of the genital tract and dorsal pancreas, precursor of the pancreatic body and tail, are adjacent to one another during embryogenesis. This theory is consistent with the finding that most MCNs are located in the body and tail of the pancreas; however, up to 5–10% of MCNs are located in the head of the gland. MCNs were long thought to be premalignant lesions that always mandated resection. The current literature sets the risk of invasive carcinoma anywhere between 6 and 36%. Given the relatively low morbidity associated with a left-sided pancreatic resection, many continue to advocate an aggressive surgical approach [7], although others have proposed a selective approach to these tumors based on size and radiographic features [8,9].

The primary gross architectural feature differentiating MCN and IPMN is pancreatic ductal involvement. By definition, IPMN are associated with the pancreatic ductal system, and MCN do not contain ductal epithelium. Pancreatic cyst fluid analysis has been shown to help differentiate mucinous from non-mucinous lesions using a CEA cutoff of 192 mg/dl [10], and this is now a routinely adopted test. Similarly, cyst fluid amylase has been used to define involvement with the pancreatic ducts. This analyte appears to have the greatest diagnostic use when distinguishing between pancreatic pseudocysts and cystic neoplasms including IPMN and MCN [11]. Several studies do not show any difference in utilizing cyst fluid amylase in differentiating MCN and IPMN [12,13]; however, these studies do not separate main duct IPMN from branch duct lesions. Smaller series do note very high amylase levels in the cystic aspirate are associated with main duct IPMN [14].

Over 1000 cases of MCN have been reported in the literature. Of these, the vast majority have been single lesions, and only a handful of isolated cases with multiple – usually double – lesions exist. Four such cases of double synchronous lesions have been described [[15], [16], [17], [18]], along with one case in which four cystic lesions histologically consistent with MCN were found in a male [19]. Only one case of diffuse MCN has been previously described in the literature [20]. Here, Chen et al presented a 47-year-old female with diffuse involvement of the pancreas by multifocal cysts most consistent with MCN. However, the authors were not able to demonstrate the presence of ovarian-type stroma, required for the diagnosis of MCN, in their pancreatectomy sample, despite immunohistochemical staining showing positivity for estrogen receptor and smooth muscle actin. Therefore, the authors conclude that their case’s features are most consistent with MCN, without being able to reach a definitive histological diagnosis based on WHO criteria. For that reason, this is to our knowledge the first case report of diffuse histology-proven MCN of the pancreas.

Other differences exist between these two cases. While our aspirate was able to demonstrate the presence of extracellular mucin and an elevated CEA level, both used as markers for mucinous lesions, theirs was non-diagnostic with a CEA level of zero and no mucin found. Interestingly, our cyst aspirate also showed high levels of amylase, which usually indicates ductal communication that also seemed to be present on imaging. Although MCNs are usually defined by their absence of communication with the pancreatic ducts, one of their distinguishing features from IPMNs, two cases of histology-proven MCN with ductal communication have been previously described [21,22]. To our knowledge, this is the third reported case of MCN with communication to the pancreatic ducts.

A multi-disciplinary approach should be used for the management of pancreatic cystic disease. Pseudocyst treatment has been heavily influenced by Bradley et al., who suggested that cysts larger than 6 cm in size persisting longer than 6 weeks should undergo enteric drainage [23]. This approach has since been altered to focus on those cysts that enlarge or cause mass effect. Once thought to be rare entities, cystic neoplasms are now far more common – perhaps due to higher quality imaging and diagnostic modalities. Although the majority of pancreatic cysts found in the aftermath of episodes of pancreatitis are pseudocysts, cystic neoplasms should be part of the differential. In fact, pancreatitis is a common feature when managing IPMN. Given our patient’s ongoing abdominal pain and pancreatic head calcifications, it was reasonable to surmise that her symptoms were due to chronic pancreatitis with a large pancreatic pseudocyst and that she should undergo a gastro-cystic drainage procedure. However, the unusual appearance of these cysts during radiological review with high quality imaging seemed to indicate a multifocal cystic neoplasm. Cyst fluid analysis actually suggested this lesion to be a mixed duct IPMN given its high amylase (consistent with main pancreatic ductal involvement) and CEA (consistent with mucinous neoplasm) content. After multidisciplinary review, the patient was advised to undergo a total pancreatectomy. On pathological assessment of the pancreas, the gland’s architecture had been essentially replaced by cystic disease. The dominant cyst in the pancreatic neck was a MCN (Fig. 3). Communication with a nearby pseudocyst and by extension with the main pancreatic duct likely contributed to the very high fluid amylase content obtained during endoscopic cyst aspiration. Numerous case reports have demonstrated adverse outcomes when mucinous neoplasms are treated with drainage procedures [24,25].

In conclusion, we here present an unusual case of a patient with diffuse multifocal pancreatic MCN in conjunction with pseudocysts in the setting of chronic pancreatitis. The differentiation of a cystic neoplasm from a typical cyst is fundamental in determining the appropriate treatment for these lesions. Our case highlights the importance of multidisciplinary review of these complex patients. We believe our case to be the first such report of diffuse MCN.

## Sources of funding

None.

## Ethical approval

Study is exempt from ethical approval in our institution.

## Consent

Written informed consent was obtained from the patient for publication of this case report and accompanying images. A copy of the written consent is available for review by the Editor-in-Chief of this journal on request

## Author contribution

Clara Nicolas – data interpretation, writing the paper.

Sameer Al Diffalha – study concept or design, data collection and interpretation.

Sushanth Reddy – study concept or design, data interpretation, writing the paper.

## Registration of research studies

Not registered – not first-in-man case report.

## Guarantor

Sushanth Reddy.

## Provenance and peer review

Not commissioned, externally peer-reviewed.

## Declaration of Competing Interest

None.
